# Notable Programs in Neurotology Series: The Otology Group at Vanderbilt University Medical Center

**DOI:** 10.1097/ONO.0000000000000003

**Published:** 2021-09-22

**Authors:** Elizabeth L. Perkins, David S. Haynes

**Affiliations:** Department of Otolaryngology/Head and Neck Surgery, Vanderbilt University Medical Center, Nashville, Tennessee.

## EDITORS’ INTRODUCTION

In the early years of its publication, the *American Journal of Otology* published articles featuring some of the most notable otology and neurotology programs in the country and the world. Over the years that journal has grown, changed names, and developed a second (fully open access) journal. At the same time, the subspecialties of *Otology and Neurotology* have changed and grown. With the advent of more formal certification and the need for an increasing number of specialty trained surgeons as well as other hearing, balance, and skull base professionals, the number of notable programs has dramatically increased both inside and outside the United States. Each of these programs is dedicated to providing quality patient care, performing cutting edge research, and training the next generation of both surgeons and scientists that will help lead our specialty in the future. As we launch the newest addition to the family of journals that started with the *American Journal of Otology*, we wanted to revive the tradition that started with the *American Journal of Otology*. The Journal’s editorial board has formed a special group of individuals to consider which programs to highlight with each issue of the journal. Programs can be featured for a variety of achievements in the past or in the present and can be self-nominated or nominated by the committee. As we launch this series, the obvious first choice was to start where this all started, and it is our pleasure to begin this series with a look at the birthplace of the *American Journal of Otology*. We hope our readers find these historical looks insightful but also utilize these historical profiles as a method to help our current and future programs to evolve into even better institutions for future generations.

## HISTORY

**FIG. 1.**
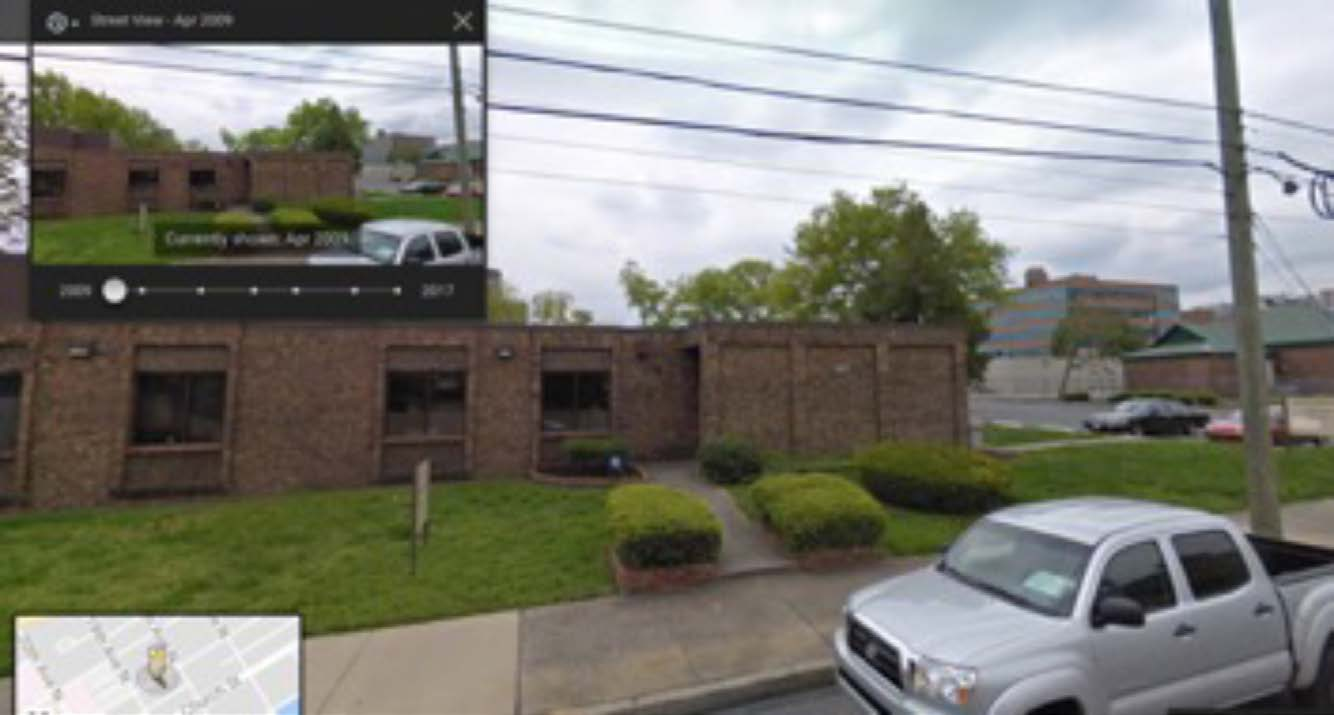
The small building of which The Otology Group was founded on State Street, Nashville, TN, by Dr Michael E. Glasscock III, M.D., in 1970.

The Otology Group was founded in a small building in Nashville, TN, on State Street (Fig. 1), as a private practice by Michael E. Glasscock III, M.D. (1933–2018) in 1970. Nearly 51 years later, The Otology Group at Vanderbilt celebrates its anniversary as one of the largest, most innovative centers for skull base surgery, speech and hearing sciences, and neurotologic education. The historic reputation of The Otology Group of Vanderbilt transcends generations of fellows and faculty members and the group remains deeply rooted in the same fundamentals and principles from which it was founded.

**FIG. 2.**
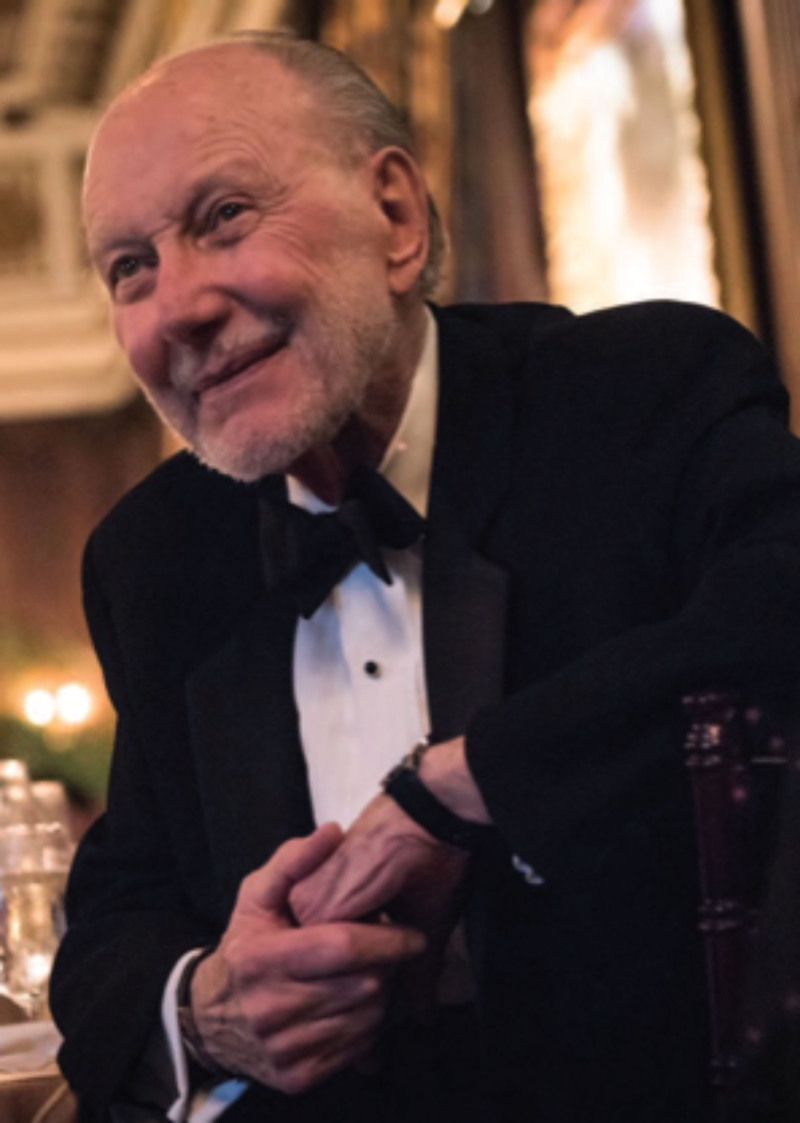
Michael E. Glasscock III, M.D. (1933–2018).

Michael E. Glasscock III, M.D. (Fig. 2) was a world-renowned clinician dedicated to patient and physician education (1). In 1980, He founded the *American Journal of Otology*, now known as *Otology and Neurotology*. He also founded the nonprofit organization, the EAR Foundation, dedicated to educating patients and physicians on ear and hearing disorders. In his career, he published nearly 250 peer-reviewed articles and was the editor of *Surgery of the Ear*. In conjunction with his partner, C. Gary Jackson, M.D., they possessed a passion for educating fellows and this passion continues to resonate within the fellowship and will do so for years to come. Dr Glasscock had sincere respect for his patients and strived to provide them with unrivaled state-of-the-art care. He was known for “doing things my way,” and such mentality (and bravado) paved the way for center that exists today.

**FIG. 3.**
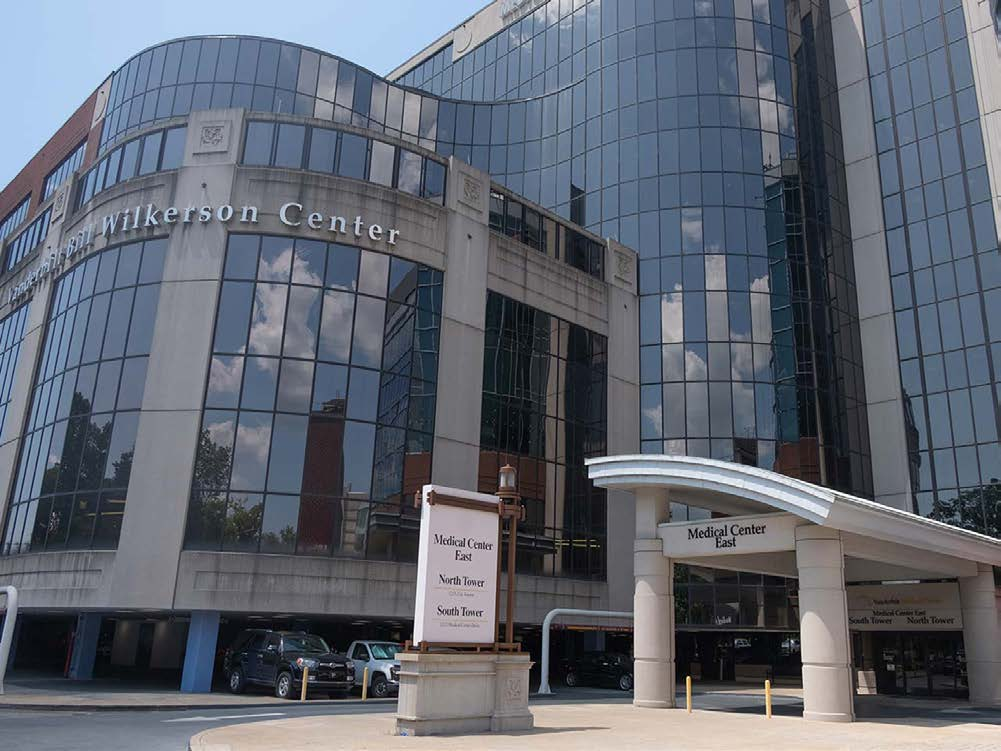
The Vanderbilt Bill Wilkerson Center as it exists today and the home of The Otology Group at Vanderbilt, The Departments of Otolaryngology and Hearing and Speech Sciences, The Mama Lere Hearing School, The Pi Beta Phi Rehabilitation Institute, Cochlear Implant Research Laboratory, The Temporal Bone Lab, The Balance Disorders Lab, and National Center for Childhood Deafness and Family Communications.

The Vanderbilt Bill Wilkerson Center (Fig. 3) was founded in 1951 by Dr Wesley Wilkerson, and named in honor of his son, Bill Wilkerson who was killed in Germany in 1945 during WWII. Wesley Wilkerson was an otolaryngologist in Nashville with a passion for pediatric hearing loss and chartered the Tennessee Hearing and Speech Foundation. In 1997, the Bill Wilkerson Center merged with Vanderbilt University Medical Center (VUMC). The Center was under the leadership for many years by Freeman O’Connell, Ph.D., then Fred Bess, Ph.D. (1978–2009) and is now under the leadership of Anne Marie Tharpe, Ph.D. (2009–current). Partnered with the Vanderbilt Department of Otolaryngology, the combined departments are now known as the Vanderbilt Bill Wilkerson Center for Otolaryngology and Communication Sciences. The Department of Otolaryngology, Head and Neck Surgery was reorganized in1985 and was led by Robert H. Ossoff, M.D. (1985–2009), Roland Eavey, M.D. (2000–2021) and is now under the leadership of Eben Rosenthal, M.D. (2021–present). The Department is ranked highly by external rankings nationwide and holds a strong reputation in both clinical care, research, and resident/fellow training (2). In 2004, the private practice which Mike Glasscock founded, The Otology Group, merged with the Vanderbilt Department of Otolaryngology, forming what is now known as The Otology Group of Vanderbilt. Within the Center is housed, in addition to The Otology Group of Vanderbilt, the Departments of Otolaryngology and Hearing and Speech Sciences, The Mama Lere Hearing School, The Pi Beta Phi Rehabilitative Institute, The Temporal Bone Lab, The Cochlear Implant Research Lab, The Balance Disorders Clinic, and The National Center for Childhood Deafness and Family Communication.

## CLINICAL ENTERPRISE

Under Dr Glasscock III, M.D.’s guidance, The Otology Group evolved into one of the world’s most preeminent centers for skull base, cochlear implants, and ear disorders. The group is now led by David S. Haynes, M.D., M.M.H.C., a direct trainee of Glasscock III, M.D. The group is composed of 5 additional faculty and 2 nurse practitioners, including Robert Labadie, M.D., Ph.D., Marc Bennett, M.D., M.M.H.C., Matthew O’Malley, M.D., Kareem Tawfik, M.D., Elizabeth Perkins, M.D., Ken Watford, D.N.P., A.P.R.N., and Emily Brignola, D.N.P., A.P.R.N., F.N.P.-C. As one of the oldest and largest neurotology practices in the country, the clinicians strive to provide innovate, evidence-based, and honorable patient care.

The Otology Group at Vanderbilt clinical practice involves a wide range of surgical approaches and techniques aimed at providing care for the most complex tumors and ear-related disorders. This includes advanced approaches to the skull base, cochlear implantation, otologic surgery, repair of cerebrospinal fluid leaks, and surgical treatment of balance disorders including superior canal dehiscence and Meniere disease. VUMC was one of the first centers to adopt endoscopic ear surgery (led by Alejandro Rivas, M.D., now at Case Western Reserve University) and to begin an annual endoscopic ear course, educating our residents, fellows, and other otolaryngologists on the new techniques of endoscopic ear surgery.

The Otology Group at Vanderbilt encompasses the renowned Skull Base Center and Hearing Implant Program, a premiere destination for patients from all over the world. The Skull Base Center upholds a strong and collaborative relationship with The Department of Neurological Surgery including neurosurgeons, Reid C. Thompson, M.D. (Chair), Lola B. Chambless, M.D., and Peter J. Morone, M.D., M.S.C.I. The Skull Base Center and Hearing Implant Programs provide multidisciplinary, coordinated care, allowing for a large number of patient visits and surgical procedures, while providing the highest quality patient care. We also have a monthly multidisciplinary aural atresia clinic attended by audiology, neurotology, facial plastics, and other specialists. In addition, we have multidisciplinary teams that care for Neurofibromatosis Type II, and pediatric hearing loss, led by Kareem Tawfik, M.D. and Elizabeth L. Perkins, M.D., respectively. In 2020, The Otology Group of Vanderbilt performed over 400 skull base consultations and 140 skull base procedures despite the challenges brought by a global pandemic. Nearly 1500 patients were evaluated in the Balance Disorders Lab in 2020. In addition, 300 patients underwent cochlear implantation both in 2019 and in 2020, making VUMC the largest cochlear implant center in the United States and one of the largest in the world.

## RESEARCH

**FIG. 4.**
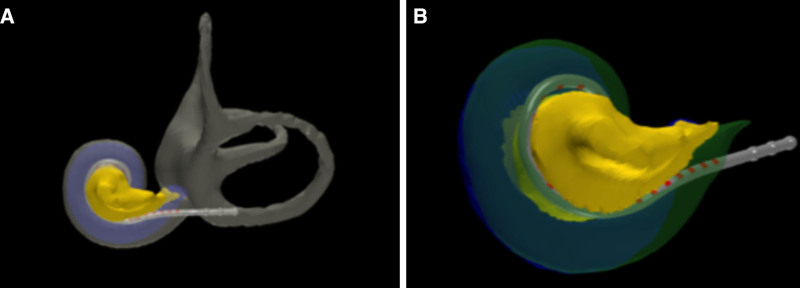
Computed tomography (CT) scan analysis of electrode array placement following cochlear implantation. *A*, CT scan analysis with a perimodiolar electrode array including the cochlear, vestibule, and semicircular canals. *B*, Closer visualization of the electrode array and cochlea allows for assessment of the electrode array position with the modiolus (yellow) and scala tympani (blue) and scala vestibuli (green).

The Cochlear Implant Research Laboratory includes principal investigators René Gifford, Ph.D. and Robert Labadie, M.D., Ph.D., and other investigators who continue to lead in international cochlear implant research. Research at VUMC ranges from reports on clinical and surgical effectiveness and quality outcomes reviews, to randomized controlled trials, such as the impact of image-guided cochlear implant programming on pediatric speech development. Research is supported from departmental resources, industry sponsored trials, and extramural funding (3–6). Our departments are ranked no. 2 in the nation in National Institute of Health (NIH) research and recent recipients of a $3.1 million NIH grant. Our recent NIH funding includes analysis of pre- and postinsertion computed tomography scanning to accurately estimate the electrode position which is used in preoperative planning and postoperative cochlear implant mapping (Fig. 4A, B) (7,8).

A highly integrated, multidisciplinary team consisting of audiologists, speech language pathologists, hearing scientists, computer science engineers, and surgeons has been facilitated by institutional resources. VUMC sits on the same campus as Vanderbilt University, allowing close collaboration with other departments primarily Department of Speech and Hearing Sciences, Electrical Engineering and Computer Sciences. Vanderbilt Institute for Surgery and Engineering arose from these collaborations and continues to develop innovative solutions for surgical procedures including skull base and cochlear implant procedures. Our colocation of services has also allowed for extensive optimization of clinical delivery that includes same-day work up and surgery for cochlear implants and bundled pricing for cochlear implant services, the first in the world (9). This collaborative environment motivates basic discoveries which can then be clinically translated to improve individual outcomes. Efforts to use our electronic medical record and artificial intelligence to better coordinate clinical care and discovery is being explored by Matthew O’Malley, M.D., and will help to define our future.

## EDUCATION

**FIG. 5.**
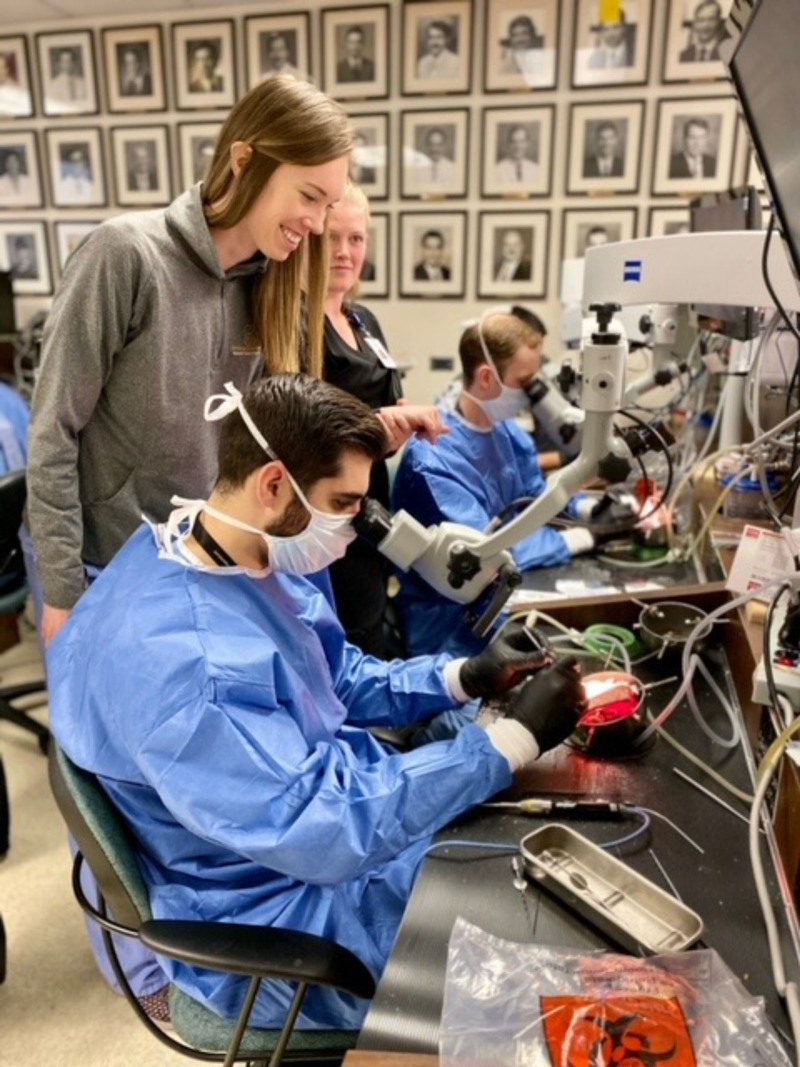
The VUMC temporal bone lab resides in the Bill Wilkerson Center. The group hosts an annual temporal bone course, with nearly 300 participants in the virtual course in Fall 2020. VUMC indicates Vanderbilt University Medical Center.

The Vanderbilt Bill Wilkerson Center is not only home to the Otology Group of Vanderbilt clinical practice but also houses our Temporal Bone lab (Fig. 5) and our research facilities. The Otology Group of Vanderbilt accepts 1 fellow per year into the neurotology program for a complement of 2 who join the neurotology service with 3 otolaryngology residents. Through the generous support of Michael E. Glasscock III, M.D. and Mr Herbert O. Christopher, the fellowship is enabled to pursue educational, academic, and research endeavors that will improve patient outcomes. Keeping with the theme of physician education, the first temporal bone lab was built in 1970 by Michael E. Glasscock III, M.D., with Jack Urban. The Otology Group of Vanderbilt continues to host multiple workshops and continuing medical education (CME) courses. This includes the annual temporal bone course, with the 85th version being held in the Fall of 2021 and the 7th annual Endoscopic Ear Course held next spring. In addition, an annual CME Otolaryngology course is held in Vail, CO, providing neurotologic education to neurotologists, otolaryngologists, and resident trainees. We also host an in-house resident/fellow temporal bone and endoscopic courses spanning 6 weeks led by Marc Bennett, M.D.

Fellow and resident education is ubiquitous, with weekly grand rounds on Friday mornings and weekly dedicated neurotology lectures on Thursday mornings led by prominent speakers within the field. Stepping beyond medical education, a leadership course is instituted throughout the year covering topics such as operations, cost accounting, organizational structure, strategy, and leadership (10). Multidisciplinary care is executed through many conferences for both skull base and cochlear implant recipients. The cochlear implant conference is held monthly and attended by audiologists, speech language pathologists, surgeons, social workers, administrative team members, implant research team members, and the cochlear implant coordinator. The monthly skull base conference is attended by neurotology faculty and fellows, neurosurgeons, radiologists, radiation oncologists, skull base coordinators, and research team members.

**FIG. 6.**
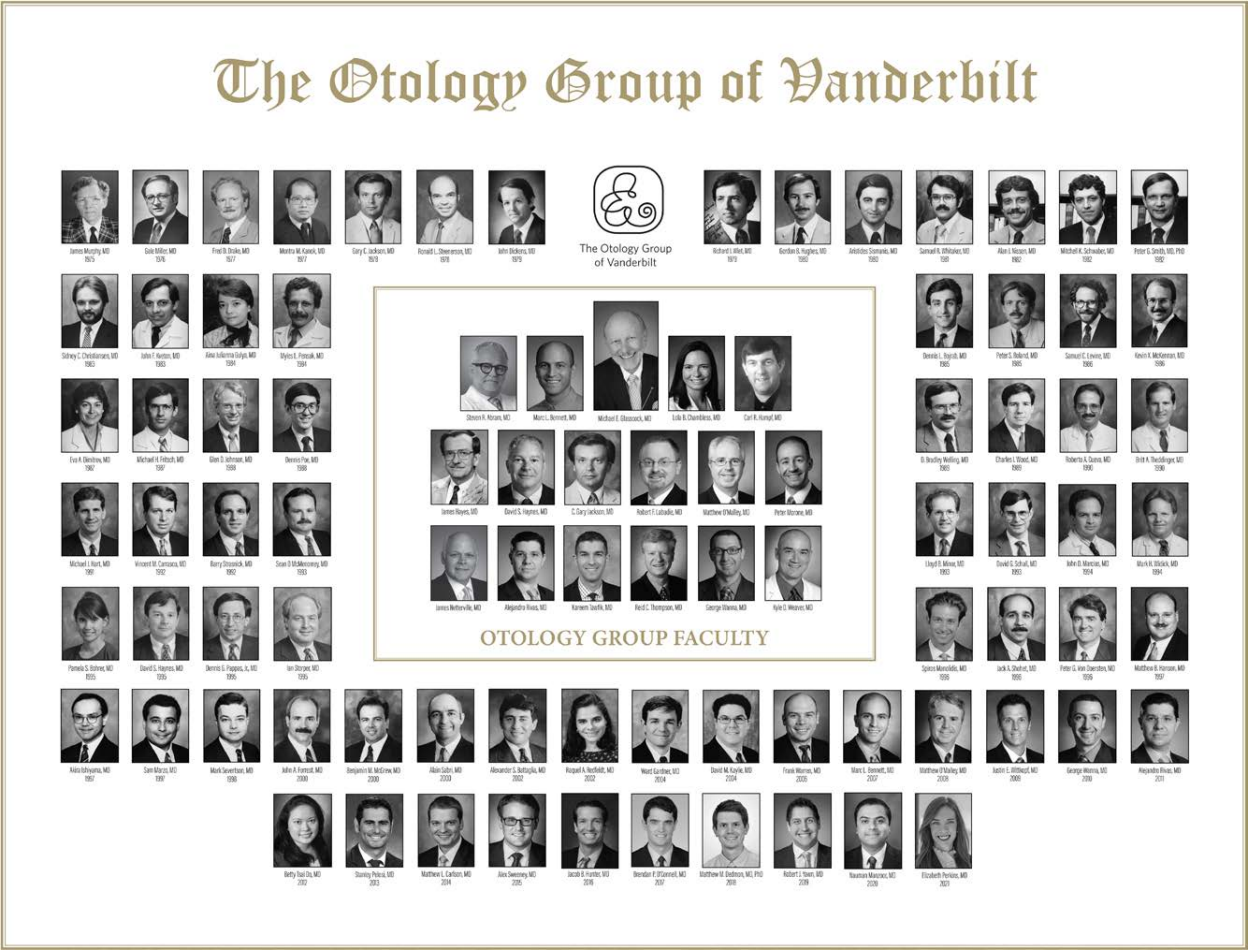
The Otology Group will graduate its 73rd fellow in the upcoming year, as shown in this group composite. In the center of the composite proudly pictures the faculty mentors and Michael E. Glasscock III, M.D.

Keeping with the Glasscock scholarly mission, The Otology Group at Vanderbilt takes considerable pride in the education of fellows and residents. This education begins with otologic surgery but transcends into every aspect of being a clinician and a leader. Fellows are “raised” to emulate the Glasscock mentality of prioritizing patient care and practicing with honor and integrity. Previous graduates and current fellows are customarily found at the podium at national and international conferences (Fig. 6) with nearly 30 to 34 peer-reviewed publications per year and over 90 scientific presentations at meetings, courses, and workshops annually. Prior fellows have held leadership positions in multiple international organizations including the American Neurotologic Society, the American Otologic Society, Triological Society, The Collegium Oto-Rhino-Laryngologicum Amicitiae Sacrum, the American Cochlear Implant Alliance, and many others. The fellows graduate with extensive operative and clinical experience, moving on to flourish within the field as division directors and chairpersons, academic deans, successful private practitioners, and academic leaders. This year, the Otology Group at Vanderbilt will proudly graduate its 73rd fellow in neurotology. Built on the legacy of Michael E. Glasscock III, M.D., the Otology Group at Vanderbilt will continue to strive for excellence in research, education, leadership training, and constantly seek innovation and improvement in patient care.

As a rapidly growing department, The Otology Group at Vanderbilt is committed to providing care for all populations and modeling diversity. Such vision is facilitated through expanding care to remote populations involving telehealth, expedited and personalized care, and physician outreach. The group fosters diversity and equality through onboarding international faculty and fellows who reflect the increasingly diverse society we serve.

## CONCLUSION

The Otology Group at Vanderbilt has strived from its inception to provide the highest quality patient care and to train the best neurotologic surgeons to become leaders in our field. We aspire to discover and provide novel and innovative treatments that will improve the lives of our patients. Education of patients, practicing physicians, and physicians in training remains an essential component of our group. As we celebrate our 51st year, our values remain stronger than ever.
